# Supplementary material for: Genetic Relatedness, Antibiotic Resistance, and Effect of Silver Nanoparticle on Biofilm Formation by Clostridium perfringens Isolated from Chickens, Pigeons, Camels, and Human Consumers
Source: Vet Sci. 2022 Mar 2;9(3):109. doi: 10.3390/vetsci9030109 (PMC8949260; doi:10.3390/vetsci9030109)
Supplement: Supplementary file 1 [file vetsci-09-00109-s001.zip › vetsci-1597109-supplementary.pdf]

Supplementary Table S1. Average OD620 of biofilm production by *Clostridium perfringens* in the presence of different AgNPs concentrations

Isolate	AgNPs Concentration (µg/ml)										
	5	10	20	30	40	50	60	70	80	90	100
CT4	0.851	0.788	0.721	0.653	0.602	0.551	0.493	0.4231	0.311	0.256	0.2123
CM48	0.911	0.779	0.713	0.668	0.621	0.536	0.462	0.4021	0.293	0.247	0.2124
PT5	0.904	0.775	0.735	0.658	0.629	0.551	0.454	0.4233	0.281	0.2462	0.2241
CF8	0.881	0.789	0.722	0.671	0.643	0.5502	0.511	0.4313	0.311	0.253	0.2243
CA49	0.893	0.767	0.723	0.659	0.622	0.538	0.512	0.4223	0.3102	0.261	0.2326
HU38	0.874	0.776	0.7013	0.669	0.627	0.5504	0.512	0.4203	0.3105	0.257	0.2251

CT: Chicken intestine, CM: Chicken meat, PT: Pigeon intestine, CF: Camel feces, CA: Camel meat, HU: Human stool.

Supplementary Table S2. Percent inhibition of biofilm formation by various concentrations of AgNPs against *Clostridium perfringens*. The absorbance was measured at 620 nm for the quantification of biofilm formation.

Isolate	AgNPs Concentration (µg/ml)										
	**5**	**10**	**20**	**30**	**40**	**50**	**60**	**70**	**80**	**90**	**100**
**CT4**	29.08333	34.33333	39.91667	45.58333	49.83333	54.08333	58.91667	64.74167	74.08333	78.66667	82.30833
**CM48**	24.08333	35.08333	40.58333	44.33333	48.25	55.33333	61.5	66.49167	75.58333	79.41667	82.3
**PT5**	24.66667	35.41667	38.75	45.16667	47.58333	54.08333	62.16667	64.725	76.58333	79.48333	81.325
**CF8**	26.58333	34.25	39.83333	44.08333	46.41667	54.15	57.41667	64.05833	74.08333	78.91667	81.30833
**CA49**	25.58333	36.08333	39.75	45.08333	48.16667	55.16667	57.33333	64.80833	74.15	78.25	80.61667
**HU38**	27.16667	35.33333	41.55833	44.25	47.75	54.13333	57.33333	64.975	74.125	78.58333	81.24167

CT: Chicken intestine, CM: Chicken meat, PT: Pigeon intestine, CF: Camel feces, CA: Camel meat, HU: Human stool.
